# Obituary

**Published:** 1888-06

**Authors:** 


### Obituary.

### EDWARD S. DUNSTER, M.D.

Dr. E. S. Dunster, A.M., M.D., Professor of Obstetrics and diseases of women and children and Clinical Gynæcology in the University of Michigan, died May 3d, 1888. Dr. Dunster was a descendant of Henry Dunster, the first president of Harvard University, and was born at Springvale, Maine, in 1837. He took the degree of A.M. at Harvard in 1846 and that of M.D. at the College of New York in 1849. He was Demonstrator of Anatomy at Dartmouth College from 1849 to 1860; Surgeon in the United States Army from 1861 to '66; was Professor in the University of Vermont from 1866 to '75; and Editor of the New York *Journal* from 1866 to '71. In 1877 he was elected to a professorship in the University of Michigan, which position he held at the time of his death. Dr. Dunster's health had been precarious for a year. He was an indefatigable worker, and perhaps to this fact was due in some measure at least his breaking down. His death is a great loss to the Medical Department of the University, and indeed to the University as a whole, for he had a great interest in all its work. He was one of the finest lecturers to whom we have ever listened. Men of such ability can ill be spared from any institution. The Dental Department will feel his loss very greatly, for to him its students were always much attached. His death is the third of the prominent teachers of this department of the University within the last few months. Never before was such a breach made in so short a time in the University.

